# Telephone‐based behavioral activation with mental imagery for depression: A pilot randomized clinical trial in isolated older adults during the Covid‐19 pandemic

**DOI:** 10.1002/gps.5646

**Published:** 2021-11-10

**Authors:** Johnny Pellas, Fritz Renner, Julie L. Ji, Mattias Damberg

**Affiliations:** ^1^ Department of Public Health and Caring Sciences Uppsala University Uppsala Sweden; ^2^ Centre for Clinical Research Uppsala University, Västmanland County Hospital Västerås Sweden; ^3^ Department of Clinical Psychology and Psychotherapy University of Freiburg Breisgau Germany; ^4^ School of Psychological Science University of Western Australia Perth Western Australia Australia

**Keywords:** activity scheduling, behavioral activation, depression, mental imagery, older adults, social isolation

## Abstract

**Objectives:**

To shield vulnerable persons, particularly the elderly, during the Covid‐19 pandemic governments around the world have advised to use social distancing and self‐isolation. Social isolation might put older adults at an increased risk for mental health problems such as depression. There is a need for brief, easy‐accessible psychological treatments for depressive symptoms that can be delivered remotely. The aim of this study was to investigate the feasibility, acceptability, and preliminary efficacy of telephone‐delivered Behavioral Activation with Mental Imagery (BA‐MI) for the treatment of depressive symptoms in individuals 65 years and older living in isolation during the Covid‐19 pandemic.

**Methods:**

In this open‐label pilot randomized clinical trial, *N* = 41 individuals aged 65 years or older with clinically significant symptoms of depression were randomly assigned to either a BA‐MI treatment condition, or an Attention‐Assessment control condition delivered over the telephone over a 4‐week period.

**Results:**

Depressive symptoms decreased more in the treatment condition compared to the control condition. At post‐treatment, 2 out of 16 participants in the treatment condition met diagnostic criteria for depression compared to 9 out of 13 in the control condition. Most participants in the treatment condition were satisfied with the treatment and few adverse effects were observed.

**Conclusions:**

This pilot study suggests that behavioral activation with mental imagery delivered over the telephone is feasible, acceptable, and potentially efficacious for the treatment of depressive symptoms in older individuals living in isolation. Replication in larger samples is needed.

## Key points

- The Covid‐19 pandemic has exacerbated the need for brief, accessible psychological treatments for depressive symptoms that can be remotely delivered to isolated older adults.
- This pilot RCT investigated the feasibility and preliminary efficacy of telephone‐based Behavioral Activation with Mental Imagery (BA‐MI) for depression in this sample.
- BA‐MI led to a greater decrease in depressive symptoms and increases in behavioral activation in the treatment group compared to the control group.
- BA‐MI may help to alleviate depressive symptoms in isolated older adults.

## 1 INTRODUCTION

The Severe Acute Respiratory Syndrome Coronavirus 2 (SARS‐CoV‐2) causing the Coronavirus disease 2019 (Covid‐19) became a pandemic in the year 2020 and into 2021. Covid‐19‐related mortality rates were the highest in older adults particularly those aged 70 years and above. In Sweden, between April and October 2020, the government urged people 70 years and older to limit physical contact with other people and to stay at home as much as possible, a strategy called social distancing.^1^ While these measures are important to shield vulnerable individuals, mental health experts have raised serious concerns over deteriorating mental health during and after the pandemic,^2^ particularly among older adults.^3^ Indeed, social isolation is related to an increase risk for depression and anxiety disorders among older adults.^4^ Thus there is a need to develop and evaluate scalable psychological interventions for mental health problems for this patient group.

Evidence from previous epidemics and the current pandemic indicates that there is an urgent need for effective remotely delivered psychological interventions aimed at improving mental health among older individuals.^5^ Research on the SARS epidemic in 2000 indicates that quarantine and isolation may lead to an increase in mental health conditions, such as depression.^6^ Depression in older adults increases the risk of mortality and morbidity,^7^ reduces quality of life,^8^ and leads to functional impairments.^9^ A study from Hong Kong during SARS found a nearly 32% increase in suicide rates among older individuals.^10^ A recent review showed that social distancing has a negative impact on the mental as well as physical health of older individuals, including increased levels of depression, anxiety, and sleep problems.^5^ A recent study in older adults from Sweden found that half of the sample reported staying at home all of the time, and that 44.4% of females and 25.7% of males reported feeling depressed.^11^

Current evidence‐based treatments for depression in older adults include antidepressant medication,^12^ psychological interventions such as Cognitive–Behavioral Therapy (CBT),^13^ and physical activity.^14^ Previous studies have shown that the majority of older adults prefer psychological treatments to medication,^15^ which poses a challenge during the Covid‐19 pandemic as such treatments are often delivered face to face. Psychological treatments delivered via the Internet are as efficacious as face to face,^16^ but only 3%–4% of the individuals 65 years and above in Sweden use digital applications instead of physical healthcare visits.^17^ CBT has been shown to work when delivered via the telephone^18^ and has proven to be feasible for older adults,^19^ and one study showed that it may even result in lower attrition compared to face‐to‐face psychotherapy.^20^ Due to the wide availability and accessibility of telephones, telephone‐based psychological treatment could be an optimal intervention for older adults, particularly during pandemics.

Behavioral Activation (BA) is a brief psychological treatment that aims to (1) increase engagement in adaptive activities, (2) decrease engagement in activities that maintain depression or increase risk for depression, and (3) solve problems that limit access to rewarding activities or that maintain aversive control.^21^ Common BA strategies are self‐monitoring of activities and mood, planning and scheduling of activities in line with the patients' needs and goals, and problem solving.^21^, ^22^ BA is effective in reducing depressive symptoms^23^, ^24^ and there is emerging evidence suggesting that BA is also an effective intervention for older adults.^25^ BA can be delivered in a brief format,^26^, ^27^ and has been shown to be feasible for older individuals in as few as four sessions.^28^, ^29^ However, depression is often characterized by low motivation and a lack of energy, which might make it more difficult for patients to initiate engagement in scheduled activities thereby reducing the efficacy of BA interventions. Thus identifying interventions that directly target these motivational difficulties could provide an additional route to promote greater behavioral engagement in scheduled reward activities. One way to facilitate motivation for planned activities might be via prospective mental imagery.

Mental imagery refers to the representation and experience of sensory information without external input.^30^, ^31^ By drawing upon prior knowledge and experiences, mental imagery can amplify the anticipation of reward‐related emotions.^32^, ^33^ Anticipation of the pleasant and rewarding consequences of future behavior, in turn, predicts reward motivation and reward‐motivated behavior.^34^, ^35^, ^36^ Indeed, it has been shown that simulating engagement in scheduled pleasant and rewarding activities via mental imagery can increase motivation to engage in these activities.^37^, ^38^, ^39^ Similar mental imagery interventions have successfully been used by older individuals.^40^

The aim of this open‐label pilot randomized clinical trial was to investigate the feasibility and preliminary efficacy of a brief telephone‐delivered BA intervention, focused on activity scheduling augmented with mental imagery, for the treatment of depressive symptoms in individuals 65 years and older in isolation during the Covid‐19‐pandemic. We hypothesized that participants randomized to the active treatment condition would show a stronger decrease in depressive symptoms (primary outcome) and improve more on secondary outcomes, compared to participants randomized to an attention‐assessment control condition.

## 2 METHODS

### 2.1 Design

The present study was an open‐label pilot randomized clinical trial (*N* = 41) with two conditions: (1) telephone‐delivered Behavioral Activation with Mental Imagery (BA‐MI), and (2) Attention‐Assessment Control with weekly follow‐up calls. This was a single‐site trial, with all participants recruited from one county in Sweden and treated through the Adult Psychiatric Clinic at Västmanland County Hospital. The study received ethical approval by the Swedish Ethical Review Authority (2020‐02079) and all participants provided written informed consent. The study was preregistered with ClinicalTrials.gov (NCT04508868). Note that the initial pre‐registered plan was to conduct a full‐scale trial, but due to changes in self‐isolation restrictions for older individuals in Sweden from 22 October 2020, the recruitment had to be terminated early.

### 2.2 Participants

Participants were approached using advertisements in local newspapers that invited individuals aged 65 years or older living in the county of Västmanland to participate in a telephone‐based treatment of depressive symptoms. The inclusion criteria were (1) reporting clinically significant depressive symptoms as defined by scores above 12 on the Montgomery‐Åsberg Depression Rating Self‐rating Scale (MADRS‐S), and/or scores above 9 on the Patient Health Questionnaire 9‐item (PHQ‐9), and/or scores above 5 on the Geriatric Depression Rating Scale 15‐item—short form (GDS‐15), and/or via structured clinical interviews; (2) access to telephone; (3) fluent in written and spoken Swedish; (4) residing in the County of Västmanland; and (5) willing to participate in the trial. Exclusion criteria included severe depression defined by clinical diagnosis, elevated risk of suicide, current substance use disorder, current or previous manic/hypomanic episodes, current psychotic disorder, current diagnosis of dementia/major neurocognitive disorder, currently receiving psychological therapy, or currently undergoing pharmacological treatments that commenced less than 1 month ago. Participant characteristics are described in Table 1. Baseline ratings on primary and secondary outcome measures are described in Table 3.

**TABLE 1:** Baseline demographic and clinical characteristics

Baseline characteristic	Treatment group (*N* = 20)	Control group (*N* = 20)
Age, mean (SD)	75.95 (8.16)	75.15 (6.20)
Women, *n* (%)	16 (80)	17 (85)
Partner/cohabiting, *n* (%)	9 (45)	7 (35)
Psychotropic medication		
Antidepressant medication, *n* (%)	0 (0)	5 (25)
Anxiolytic medication, *n* (%)	2 (10)	2 (10)
Sleep medication, *n* (%)	5 (25)	3 (15)
Psychiatric diagnoses according to MINI^a^		
Major depression	16 (80)	14 (70)
Panic disorder	1 (5)	0 (0)
Posttraumatic stress disorder	2 (10)	0 (0)
Generalized anxiety disorder	0 (0)	1 (5)

Abbreviations: MINI, Mini International Neuropsychiatric Interview 7.0; SD, Standard deviation.

^a^ No current diagnoses of bipolar disorder, psychotic syndromes, or alcohol‐/substance use disorder since these were exclusion criteria.

### 2.3 Materials

#### 2.3.1 MINI

The Mini International Neuropsychiatric Interview 7.0 (MINI),^41^ a structured clinical interview, was used to assess the presence/absence of common psychiatric disorders at enrollment as well as assessing the presence/absence of depression post‐treatment.

#### 2.3.2 Primary outcome measure

##### MADRS‐S

The primary outcome measure was the MADRS‐S, which is a nine‐item questionnaire, used to measure severity of depression the past 2 weeks.^42^ The score ranges from 0 to 54, with higher scores indicating higher depression severity.

#### 2.3.3 Secondary outcome measures

##### GDS‐15

Depressive symptoms were also measured using the GDS‐15.^43^ The GDS‐15 is a 15‐item questionnaire used to identify depression in older individuals with scores ranging from 0‐15.

##### PHQ‐9

The PHQ‐9 is a nine‐item questionnaire used to identify depression as well as measuring severity of depression with scores ranging from 0 to 27, with higher scores indicating higher depression severity.^44^

##### GAD‐7

Anxiety symptoms were assessed with the GAD‐7, a seven‐item questionnaire used to identify generalized anxiety disorder as well as measuring severity of anxiety symptoms.^45^ The score ranges from 0 to 21, with higher scores indicating higher anxiety severity.

##### BADS‐SF

Changes in avoidance and activation was measured using the Behavioral Activation for Depression Scale—Short Form (BADS‐SF).^46^

##### WHODAS‐12

Functional impairment was assessed with the WHODAS‐12, a self‐rating scale with 12 items.^47^

#### 2.3.4 Mental imagery ability

##### Psi‐Q

Mental imagery was assessed at baseline and post‐intervention using the Plymouth Sensory Imagery Questionnaire (Psi‐Q),^48^ translated by Johanna M. Hoppe and Emily Holmes. Psi‐Q is a 35‐item questionnaire assessing the vividness of mental imagery. In this study, we used the visual subscale with five items, with a total score ranging from 0 to 50, with higher scores indicating higher imagery ability.

#### 2.3.5 Iatrogenic effects

##### NEQ

Adverse effects were measured using the Negative Effects Scale (NEQ)—short form, a 20‐item questionnaire used to assess adverse and unwanted effects of psychological treatments.^49^ In the analysis, only the items rated by the participant as probably caused by the treatment were included. Adverse effects were also monitored by the therapists throughout the interventions.

#### 2.3.6 Feasibility and acceptability

Feasibility was assessed by recording recruitment and dropout rates. Time was measured for each session, in the treatment group as well as in the control group. Acceptability was measured with the question “overall, how satisfied were you with the treatment you received?”, with ratings ranging from 1 to 5 representing “very dissatisfied” to “very satisfied”, with 3 representing a neutral alternative, “neither satisfied nor dissatisfied”.

### 2.4 Interventions

The treatment manual as well as the manual for the control group was adapted into web‐based versions on the system Entermedic. The digital manuals enabled us to upload the correct session manuals for each participant, digitalize in‐session ratings, as well as providing a checklist for each treatment component to ensure therapists adherence to the manuals, inspired by Serfaty and colleagues.^50^

#### 2.4.1 Treatment group

A treatment manual was constructed, based on two interventions: a four‐session brief BA treatment for primary care (BA‐PC) published by Funderburk and colleagues,^28^ with the addition of a mental imagery script by Renner and colleagues'^37^ sessions two and three. The interventions and patient materials were translated into Swedish by the corresponding author, and was then reviewed by two senior clinical psychologists and a primary care physician. The BA‐PC was adapted to comply with government restrictions due to Covid‐19. The session outline is described in Table 2.

**TABLE 2:** Session components in the treatment group

Session no	Session components
1	Assess mood and suicide risk
	Provide psychoeducation about depression
	Provide treatment rationale for Behavioral Activation (BA)
	Provide rationale and instructions for activity log
	If highly motivated for change, plan activity for coming week
2	Follow‐up on mood and suicide risk
	Review activity log
	Discuss life goals and values
	Plan activities aligned with life goals and values for coming week
	Provide rationale for Mental Imagery (MI)
	Go through MI exercise for one of the planned activities
3	Follow‐up on mood and suicide risk
	Review activity log
	Troubleshoot any problems carrying out activities
	Plan activities aligned with life goals and values for coming week
	Go through MI exercise for one of the planned activities.
4	Follow‐up on mood and suicide risk
	Review activity log
	Troubleshoot any problems carrying out activities
	Review treatment
	Stress the importance of continuing to engage in activities aligned with life goals and values
	Referral to additional service/s if necessary

#### 2.4.2 Control group

The control group was placed on a waiting list with weekly follow‐up calls. The manual for the control group included follow‐up on the patient's psychological symptoms, assessment of suicide risk and answering practical questions about the study. The therapists were instructed to be supportive by using validation techniques for thoughts and feelings, but not to engage in behavioral interventions such as activity planning or problem solving.

#### 2.4.3 Therapists

Five psychologists participated in the study, of which two were senior licensed psychologists and three were psychologists during their 1‐year clinical internship before license. All of the psychologists had at least basic training in cognitive behavioral therapy. They had a 1‐day training workshop which included information about and practical training in delivering the treatment manual. The therapists had a supervision session with the corresponding author every 2 weeks to resolve practical issues and troubleshoot difficult treatment situations.

### 2.5 Procedure

Interested participants contacted the research unit and received information about the study. A psychologist or research nurse screened the participants concerning age, subjective symptoms of depression, county of residence, and language comprehension. Participants who met the screening criteria were sent detailed information about the study, informed consent forms, and baseline questionnaires. Upon return of signed consent forms and baseline questionnaires, the corresponding author phoned the participant and conducted a clinical interview including a structured psychiatric interview to evaluate eligibility. Participants who met all inclusion criteria and no exclusion criteria were consecutively randomized to either treatment or control, with one weekly call for four weeks. After 4 weeks, the control group also were offered the treatment. The participants completed depression ratings after each call week 1–3, and at week 4 (post‐treatment/post‐control) they completed a more comprehensive questionnaire.

#### 2.5.1 Randomization and blinding

An independent statistician generated a 1:1, 6‐block randomization sequence. The statistician prepared sequentially numbered opaque envelopes to conceal allocation until the moment of randomization. The corresponding author was responsible for evaluating eligibility and enrolling subjects in the study groups. The baseline questionnaires were scored by a research nurse and blinded to the psychologist until after the structured clinical interview. All subsequent self‐ratings were performed by the participants at home and sent by mail, and were scored by a research nurse and thereby blinded to the psychologists. A structured clinical interview was performed at week 4/post‐treatment by the patient's therapist.

### 2.6 Measures

The MADRS‐S was assessed weekly. Acceptability and adverse effects were assessed at post‐treatment. All other measures were assessed at baseline and post‐treatment.

#### 2.6.1 Analysis plan

To test the effect of the intervention condition on the primary outcome measure, a repeated‐measures ANOVA was conducted with condition (BA‐MI vs. control) as the between subject factor and time (baseline vs. post‐intervention) as the within subject factor. Between‐groups effect sizes post‐treatment on the primary outcome measure and continuous secondary outcome measures were calculated using Hedge's *g*. Hedge's *g* was chosen as it’s more suitable for smaller samples as well as differences in sample size between groups. The magnitude of the effect sizes is interpreted as follows: in line with the recommendation for gerontological research^51^: 0.15 = small, 0.40 = medium, and 0.75 = large. Difference in the number of participants meeting diagnostic criteria for depression post‐treatment was calculated using Fisher's exact test.

## 3 RESULTS

**FIGURE 1:**
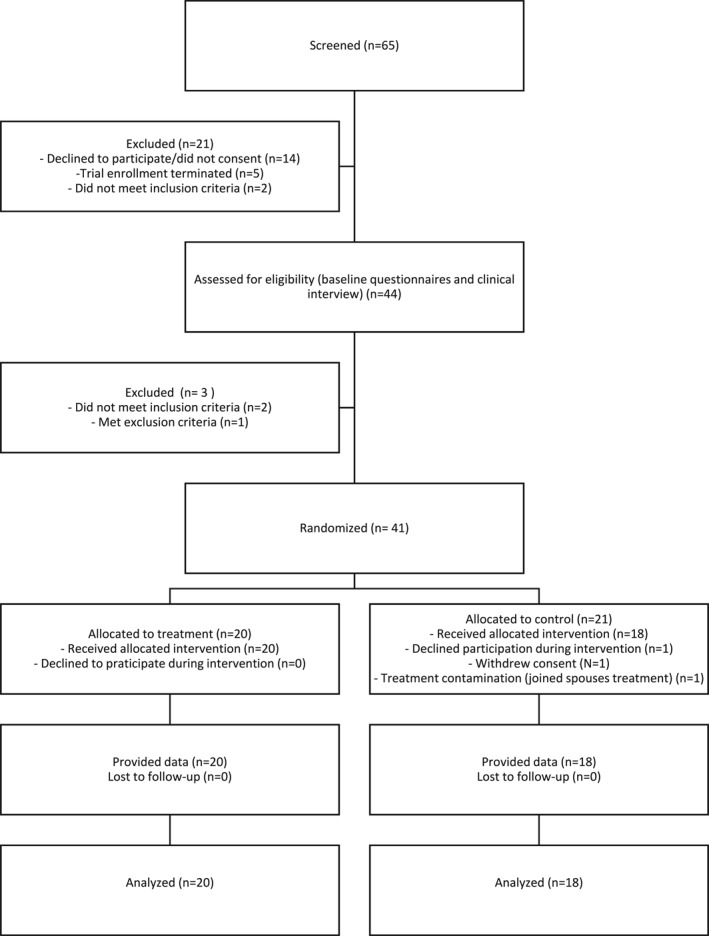
Participant flow through the trial

Recruitment took place between August and October 2020. Forty‐one participants were randomized to the intervention (*N* = 20) or the control condition (*N* = 21). Participant flow is described in the CONSORT diagram in Figure 1.

### 3.1 Feasibility and acceptability

Of 65 screened individuals, 41 (63%) were found eligible for the study and subsequently randomized. Of the 41 randomized participants, 2 dropped out and 1 was excluded during the trial, all in the control group, making the overall dropout rate 7.3%.

Mean time per session in the treatment group was 43.55‐min session 1, 43.35‐min session 2, 32.10‐min session 3, and 29.05‐min session 4. Time per session in the control group was 16.06‐min session 1, 12.61‐min session 2, 12.89‐min session 3, and 16.67‐min session 4. All participants received the treatment or control condition in line with the manual.

Eighteen out of 20 participants in the treatment group rated treatment satisfaction. Five rated themselves as very satisfied with the treatment, seven as somewhat satisfied, five as neither satisfied nor dissatisfied, none as somewhat dissatisfied, and one as very dissatisfied with the treatment.

### 3.2 Primary outcome measure

**FIGURE 2:**
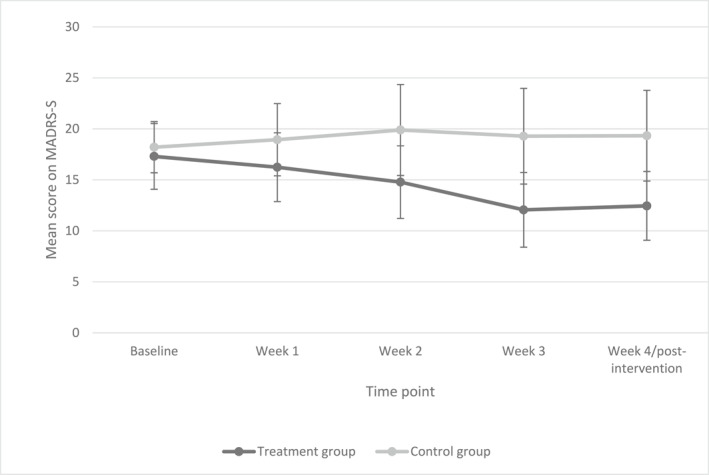
Mean depression severity over the different time points. Error bars are 95% confidence intervals. MADRS‐S, Montgomery‐Åsberg Depression Rating Self‐rating Scale

The means and 95% confidence interval for each group on MADRS‐S across the different time points are shown in Figure 2.

In the repeated‐measures ANOVA, Mauchly's test indicated that the assumption of sphericity had been violated (*x* ^2^[9] = 37.44, *p* = < 0.001); therefore, degrees of freedom were corrected using Greenhouse–Geisser estimates of sphericity (*ε* = 0.58). The results of the repeated‐measures ANOVA revealed that there was a significant Condition × Time interaction for the primary outcome measure (*F*[2.30, 73.64] = 3.71, *p* = 0.024, *η* ^2^ = 0.10), indicating a stronger decrease in depressive symptoms in the treatment group compared to the control group.

Means, standard deviations, and between‐group effect size post‐treatment on the primary outcome measure are shown in Table 3.

**TABLE 3:** Means (M), standard deviations (SD) and effect sizes of primary and secondary outcome measures at baseline and post‐intervention

	Treatment group (*N* = 20)						Control group (*N* = 18)						Effect size (g)
	Baseline			Post‐intervention			Baseline			Post‐intervention			
	*M*	SD	*n*	*M*	SD	*n*	*M*	SD	*n*	*M*	SD	*n*	
Primary outcome													
MADRS‐S	17.30	6.88	20	12.45	7.21	20	18.20	5.36	20	19.33	8.96	18	0.85
Secondary outcomes													
GDS‐15	8.45	2.76	20	6.10	3.84	20	8.05	2.14	20	8.39	2.62	18	0.69
PHQ‐9	9.65	5.06	20	6.95	5.49	19	9.75	5.59	20	10.72	7.51	18	0.58
GAD‐7	7.05	5.04	20	5.30	5.17	20	7.60	4.24	20	8.50	4.94	18	0.63
BADS‐SF	22.10	6.59	20	33.40	10.02	20	21.90	6.16	20	23.67	7.81	18	1.08
Psi‐Q	44.47	8.44	20	45.65	7.89	20	34.56	12.36	20	38.06	10.78	18	0.81
WHODAS‐12	14.35	10.41	20	10.90	8.07	20	12.50	9.03	20	13.56	10.88	16	0.28

Abbreviations: BADS‐SF, Behavioral Activation for Depression Scale ‐ Short Form; GAD‐7, Generalized Anxiety Disorder 7‐item; GDS‐15, Geriatric Depression Rating Scale 15‐item short form; MADRS‐S, Montgomery‐Åsberg Depression Rating Self‐rating Scale; PHQ‐9, Patient Health Questionnaire 9; Psi‐Q, Plymouth Sensory Imagery Questionnaire; WHODAS‐12, WHO Disability Assessment Schedule 12‐item.

### 3.3 Secondary outcome measures

Means, standard deviations, and effect sizes of secondary outcome measures at baseline and post‐intervention are presented in Table 3. Post‐intervention, 2 out of 16 participants in the treatment group still met depression criteria according to the MINI, which is lower than the control group, where 9 out of 13 participants still met criteria, but not statistically significant according to Fisher's exact test (two‐tail) *p* = 0.073.

### 3.4 Iatrogenic effects

Nineteen participants in the treatment group completed the NEQ and 4 (21%) reported adverse effects that they attributed to the treatment. The most frequent adverse effects were increased anxiety, more unpleasant feelings, that unpleasant memories resurfaced, that they didn't always understand their treatments, that the treatment didn't produce positive results, and that became afraid that other people would find out about their ongoing treatment. No serious adverse effects or serious deterioration was reported by the therapists during the interventions.

## 4 DISCUSSION

This pilot study is, to our knowledge, the first to test the feasibility and preliminary efficacy of telephone‐based BA‐MI aimed at reducing depressive symptoms in older adults during a pandemic. The treatment and study design were found to be feasible, and results indicated that a 4‐week BA‐MI intervention reduced depression symptoms (MADRS‐S) and increased behavioral activation (BADS‐SF) relative to an attention‐assessment control condition.

BA treatment aims to increase engagement in rewarding everyday activites via activity scheduling.^52^ Numerous randomized controlled trials^53^, ^54^, ^55^ and meta analyses^23^, ^24^ have shown the efficacy of BA. The evidence of BA for older adults is limited. The findings of our pilot study are in line with previous research indicating that BA might be an effective intervention for older adults,^25^ and shows promise in as few as four sessions as previously demonstrated.^28^ In addition to these previous findings, we could show that BA can be delivered remotely over the telephone and thereby reach individuals in isolation. This is crucial given that, independent of the pandemic situation, older individuals might not have access to online therapy resources.

The results of this study support the idea that mental imagery simulations of daily activities could be used to facilitate BA treatment for depression. In this study, a behavioral activation with mental imagery intervention led to a stronger increase in behavioral activation compared to an attention control condition. This is in line with previous research demonstrating a positive impact of mental imagery on behavioral activation in individuals with depression.^38^ Given the emotion amplifying properties of mental imagery, it has been argued that mental imagery might have motivational properties that facilitate engagement in planned activities. However, previous studies have not specifically combined BA procedures (activity scheduling) with mental imagery practice in a clinical sample. While our results support this idea, it should be noted that given the lack of a BA control condition without mental imagery, we cannot draw any conclusions about the added value of mental imagery in this study.

In general, the results of this study should be interpreted with caution, given the small sample size that may lead to inflated effect sizes.^56^ The number of individuals fulfilling depression criteria according to MINI decreased from 16 to 2 in the treatment group, and from 13 to 9 in the control group, which is promising given the brevity of the intervention.

Although the majority of participants (67%) in the treatment group reported being “very satisfied” or “somewhat satisfied” with the treatment, five participants reported being “neither satisfied nor dissatisfied”, and one was “very dissatisfied” with the treatment. Four participants reported adverse effects on the NEQ that they attributed to the treatment, which include experiences of anxiety, unpleasant memories, that they didn't always understand their treatment, lack of positive results, and that concerns about stigma if other people would find out about their treatment. However, no serious adverse effects or serious deterioration in symptoms was observed by the therapists during the interventions. Future studies should focus on understanding individual differences in the acceptability of telephone‐based BA treatment with mental imagery in this population. Further, many participants found the NEQ scale difficult to perform, and it would be of interest to further test the validity of this scale in a population of older adults.

Since this treatment is designed to be an accessible first‐line treatment, the inclusion criteria were broad and resulted in a heterogeneous sample ranging from subclinical depressive symptoms to moderate depressive disorder. Baseline characteristics showed that the randomization produced fairly equal groups, with the exception of ongoing antidepressant treatment, which only occurred in the control group, although there was a higher frequency of depression diagnoses in the treatment group. All patients receiving medication were on stable doses, but it is possible that this affected the results. We note that although the majority of the present study's participants (80% in the BA‐MI group, 70% in the Control group) met criteria for major depression, they were recruited from the community rather than through clinics. Thus, further research is required to evaluate the feasibility and effectiveness of the BA‐MI intervention in more severe clinical populations and those recruited via routine clinical practice.

The findings of this study, if replicated, have potential implications for the treatment of depression in older individuals in isolation. Brief telephone‐based behavioral activation could potentially be used to support older individuals to increase engagement in adaptive activities with positive effects on mood. These effects might further be facilitated by the motivation enhancing properties of mental imagery. Our findings from this initial study need replication in larger sample and future studies should include a behavioral activation without mental imagery control condition to determine the added value of mental imagery. It will be particularly pertinent for future research to evaluate the added impact of mental imagery on mood, behavior, as well as treatment acceptance. Future research should also investigate additional factors that may enhance the effectiveness of remotely delivered BA‐MI interventions. One such factor may be the addition of live face‐to‐face interaction via video link delivery. Although we are not aware of studies that have assessed the impact of adding video‐link to telephone‐based interventions, it is possible that both voice and face‐to‐face interaction with the therapist, via video link, may deliver added benefits compared to telephone voice interaction alone. However, it is also possible that video link may not be more effective than telephone delivery in older adult populations living in isolation due to technological and skills barriers.

Limitations of this study include (1) time per session differed between the treatment group and control groups. Since this was a new combination of intervention manuals, it was difficult to predict time consumption, and therefore a minimal attention‐assessment control was used. Second, the trial was open labeled, which might have influenced the results, for example, by making participants in the control group frustrated when waiting to start their treatment. However, in light of the pandemic, we thought it would be unethical not to disclose this to the participants or to use a sham condition. Third, when recommendations regarding self‐isolation restrictions changed, half of the participants were ongoing in the trial. This might have influenced the results, but since participants were included and randomized consecutively, both groups were equally influenced. Fourth, the MINI‐ interviews were conducted by the patients' therapists, which may have contributed to bias; however, the ratings were blinded to the therapists and researchers. Finally, this paper only reports the results post treatment. Future studies are needed to investigate long‐term effects.

To summarize, the Covid‐19 pandemic has highlighted the need for brief, easy‐accessible psychological treatments for depressive symptoms that can be delivered by remote, particularly for older individuals. The investigated BA‐MI treatment seems feasible, acceptable, and potentially efficacious for the treatment of depressive symptoms in older individuals in isolation during the Covid‐19 pandemic. Further studies with larger samples are needed to replicate these findings.

## CONFLICT OF INTERESTS

The authors declare no conflicts of interest.

## Data Availability

The data that support the findings of this study are available from the corresponding author upon reasonable request.
